# An unexpected new species of *Habrophlebia* from Algeria (Ephemeroptera, Leptophlebiidae)

**DOI:** 10.3897/zookeys.953.51244

**Published:** 2020-07-27

**Authors:** Lina Hanane Kechemir, Michel Sartori, Abdelkader Lounaci

**Affiliations:** 1 Laboratoire Ressources Naturelles, Faculté des Sciences agronomiques et des sciences biologiques, Université Mouloud Mammeri, Tizi-Ouzou, Algeria Université Mouloud Mammeri Tizi-Ouzou Algeria; 2 Musée cantonal de zoologie, Palais de Rumine, Place Riponne 6, CH-1005 Lausanne, Suisse Musée cantonal de zoologie Lausanne Switzerland; 3 Department of Ecology and Evolution, Biophore, University of Lausanne, CH-1015 Lausanne, Switzerland University of Lausanne Lausanne Switzerland

**Keywords:** *Habrophlebia djurdjurensis* sp. nov., taxonomy, ecology, Djurdjura, Kabylia

## Abstract

We describe a new species of *Habrophlebia*, *H. djurdjurensis***sp. nov.**, based on nymphal, imaginal, and egg stages obtained by sampling from the Great Kabylia watershed, north-central Algeria. The new species was previously identified as H. cf. fusca by Lounaci et al. 2000. *Habrophlebia djurdjurensis* is in fact more related to *H. vaillantorum* Thomas, 1996 but can be separated by characters on the nymphs and male imago. This is the fourth species of *Habrophlebia* reported from North Africa.

## Introduction

The genus *Habrophlebia* was established by Eaton (1881) and currently contains seven West Palaearctic species (Barber-James et al. 2013), which are known at all ontogenetic stages (egg, nymph, male, and female imagos), except for the nymph of *H. antoninoi* Alba-Tercedor, 2000, which remains to be described. In North Africa, until recently, three species were known: *H. vaillantorum* Thomas, 1986 (Morocco), *H. consiglioi* Biancheri, 1959 (Tunisia), and H. cf. fusca (Thomas 1998; Lounaci et al. 2000) (Algeria). The work by Benhadji et al. (2018), which looked at populations of *Habrophlebia* in northwestern Algeria (Tafna watershed) studied by Gagneur and Thomas (1988), revealed a new species (*H. hassainae* Benhadji & Sartori, 2018). It was suspected that further investigations of previously studied populations would, upon re-examination, reveal additional new species. The main objective of the present study was to investigate the populations, in north-central Algeria (Great Kabylia watershed), studied by Lounaci et al. (2000), with the help of freshly collected material at all stages.

## Material and methods

**Figure 1.**
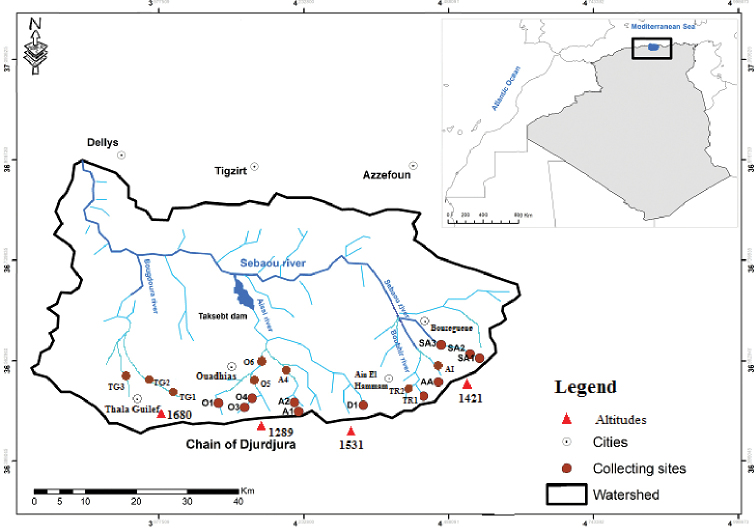
Map of Kabylia with the sampling sites.

During a period of three years, sampling sites situated in Kabylia watershed have been investigated (Fig. 1). The Kabylia of Djurdjura, located in North-central Algeria, is a mountain range whose uniqueness is the result of its climatic conditions. This region may be further subdivided into two distinct subregions : the Djurdjura, which constitutes the largest limestone massif of the Tell Atlas with peaks often exceeding 2000 m of altitude (maximum 2308 m), and the Sebaou Valley, an elongate, wide depression that is drained by Oued Sebaou, the major river of Djurdjura Kabylia. The study sites were within a drainage basin of about 4000 km^2^, which has rugged terrain with steep slopes and significant changes in altitude. The main feature of the streams is the irregularity of discharge, with episodes of floods alternating with low water periods. Water deficit in the summer induces a temporary flow regime for a large number of streams. The sampling sites belong to three different tributaries, Wadi Boubhir, Wadi Aissi, and Wadi Bougdoura (Fig. 1). They reflect the diversity of habitats and cover a wide range of ecological situations. The climate of the studied area varies from humid to sub-humid. It is characterized by a rainy season from November to May and a dry season which extends from June to October.

The average annual precipitation is around 1200 mm in the Djurdjura massif (altitude >1000 m), and around 800 mm in the Sebaou valley (Derridj 1990; Lounaci and Vinçon 2005). One of the major characteristics of the rivers studied is the significant warming of the waters in summer. In the middle and lower reaches, the maximum water temperature is high (25–30 °C), and the annual amplitudes oscillate around 20 °C. In the upper parts, the maximum temperature does not exceed 20 °C.

Nymphs were sampled either by kick sampling or by picking specimens directly from the substrate with entomological forceps. Specimens collected between 2016 and 2019 were preserved in 80% or 100% ethanol. Specimens were observed under a Leica MZ12 and M205C stereomicroscope. Mouthparts, legs, and the abdomen were mounted on microscopic slides and observed and photographed under an Olympus BX51 microscope. Photographs of the habitus were taken with a LK system (Dun Inc., Virginia). For SEM photomicrographs, eggs were dehydrated in pure ethanol, coated with 12 nm platinum; SEM work was performed at Lausanne University with a FEI Quanta FEG 250 at 10kV with a WD = 12 mm. Final figures were assembled in Adobe Photoshop CC 2018.

Material is deposited in the Museum of Zoology, Lausanne, Switzerland (MZL) and in the personal collection of the first author (KLHC).

## Taxonomy

**Family Leptophlebiidae Banks, 1900**

**Genus *Habrophlebia* Eaton, 1881**

### Habrophlebia djurdjurensis

Taxon classificationAnimaliaEphemeropteraLeptophlebiidae

Kechemir, Sartori & Lounaci sp. nov.

1098620B-12FC-5EB2-B16B-919B27C749D2

http://zoobank.org/96465B72-4E0C-4887-A067-5FEF4A56545A

Habrophlebia cf. gr. fusca Thomas 1998: 14 (partim)
Habrophlebia cf. fusca Lounaci et al. 2000: 47

#### Material examined.

***Holotype***: 1 female nymph in ethanol, (GBIFCH00672427), Algeria, Tizi-Ouzou Wilaya, Sebaou watershed, Wadi Aissi, Assif Aghalladh (O3), 36°29.28'N, 4°07.36'E, 1040 m, 09 July 2019, L.H. Kechemir coll. [MZL]. ***Paratypes***: 1 male imago (GBIFCH00672426), 15 nymphs (GBIFCH00672428, GBIFCH00672425), same data as holotype in ethanol [MZL]; 7 nymphs (GBIFCH00606856–GBIFCH00606860 and GBIFCH00673198 - GBIFCH00673199), same data as holotype on slide (Euparal) L.H. Kechemir coll. [MZL].

#### Other material (non-type material).

Algeria, Kabylia, Tizi-Ouzou Wilaya, Assif Aghalladh (O3), 36°29.28'N, 4°07.36'E, 1040 m, 24 May 2016; 26 nymphs in ethanol [KLHC] • same locality, 29 March 2017; 8 nymphs in ethanol [KLHC] • same locality, 19 April 2017; 17 nymphs in ethanol [KLHC] • same locality, 26 May 2018; 20 nymphs in ethanol [KLHC] • same locality, 07 July 2019; 7 nymphs in ethanol [KLHC].

Algeria, Kabylia, Tizi-Ouzou Wilaya, Assif Aghalladh (O4), 36°29.48'N, 4°07.49'E, 950 m, 26 May 2017, 59 nymphs in ethanol (GBIFCH00672429) [MZL]; 19 nymphs in ethanol [KLHC] • same locality, 18 March 2017; 11 nymphs in ethanol [KLHC] • same locality, 19 April 2017; 14 nymphs in ethanol [KLHC] • same locality, 27 May 2017; 28 nymphs in ethanol [KLHC] • same locality, 29 March 2018; 18 nymphs in ethanol [KLHC].

Algeria, Kabylia, Assif Aghalladh (O5), 36°30.72'N, 4°06.67'E, 500 m, 24 May 2016; 7 nymphs in ethanol [KLHC] • same locality, 18 March 2017; 5 nymphs in ethanol [KLHC] • same locality, 19 April 2017; 12 nymphs in ethanol [KLHC] • same locality, 26 May 2018; 11 nymphs in ethanol [KLHC].

Algeria, Kabylia, Assif Tamdha (O1), 36°29.98'N, 4°03.93'E, 800 m, 24 May 2016; 19 nymphs in ethanol [KLHC] • same locality, 27 May 2017; 15 nymphs in ethanol [KLHC] • same locality, 26 May 2018; 3 nymphs in ethanol [KLHC].

Algeria, Kabylia, Assif Ouadhias (O6), 36°31.88'N, 4°06.85'E, 290 m, 24 May 2016; 9 nymphs in ethanol [KLHC] • same locality, 19 April 2017; 8 nymphs in ethanol [KLHC] • same locality, 2 May 2018; 5 nymphs in ethanol [KLHC].

Algeria, Kabylia, Assif Tirourda, Com. de Tirourda (TR1), 36°29.43'N, 4°21.69'E, 1200 m, 24 April 2017; 1 nymph on slide (Euparal) (GBIFCH00673196) • same data; 4 nymphs in ethanol (GBIFCH00672430) [MZL]; 34 nymphs in ethanol [KLHC] • same locality, 26 March 2017; 10 nymphs in ethanol [KLHC] • same locality, 25 April 2017; 42 nymphs in ethanol [KLHC] • same locality, 28 May 2017; 30 nymphs in ethanol [KLHC] • same locality, 2 June 2017; 3 nymphs in ethanol [KLHC] • same locality, 30 March 2018; 8 nymphs in ethanol [KLHC].

Algeria, Kabylia, Assif Tirourda (TR2), 36°29.43'N, 4°21.53'E, 1150 m, 14 April 2016; 6 nymphs in ethanol [KLHC] • same locality, 26 March 2017; 5 nymphs in ethanol [KLHC] • same locality, 28 May 2017; 6 nymphs in ethanol [KLHC] • same locality, 30 March 2018; 12 nymphs in ethanol [KLHC] • same locality, 20 June 2018 ; 3 nymphs in ethanol [KLHC].

Algeria Kabylia, Assif Ath Atsou, Com. de Ath Atsou (AA), 36°29.71'N, 4°22.38'E, 1080 m, 14 April 2016; 3 nymphs in ethanol [KLHC] • same locality, 26 March 2017; 10 nymphs in ethanol [KLHC] • same locality, 25 April 2017; 10 nymphs in ethanol [KLHC] • same locality, 28 May 2017; 10 nymphs in ethanol [KLHC] • same locality, 30 March 2018; 15 nymphs in ethanol [KLHC] • same locality, 30 March 2018; 15 nymphs in ethanol [KLHC] • same locality, 20 June 2018; 7 nymphs in ethanol [KLHC].

Algeria, Kabylia, Assif Illithen, Com.de Illithen (AI), 36°30.41'N, 4°24.28'E, 1010 m, 24 April 2017; 2 nymphs on slide (Euparal) (GBIFCH00673194–GBIFCH00673195) ; same data 21 nymphs in ethanol (GBIFCH00672431)[MZL] • same locality, 14 April 2016; 2 nymphs in ethanol [KLHC] • same locality, 26 March 2017; 12 nymphs in ethanol [KLHC] • same locality, 28 May 2017; 27 nymphs in ethanol [KLHC] • same locality, 30 March 2018; 13 nymphs in ethanol [KLHC] • same locality, 20 June 2018; 10 nymphs in ethanol [KLHC].

Algeria, Kabylia, Assif Djemâa, Com. D’Akbil (D1), 36°30.38'N, 4°19.94'E, 900 m, 14 April 2016; 8 nymphs in ethanol [KLHC] • same locality, 28 May 2017; 23 nymphs in ethanol [KLHC] • same locality, 30 March 2018; 14 nymphs in ethanol [KLHC].

Algeria, Kabylia, Assif d’Ath Agad, Com. des Ouacifs (A1), 19 March 2017; 12 nymphs in ethanol [KLHC] • same locality, 24 May 2017; 25 nymphs in ethanol [KLHC] • same locality, 30 April 2018; 22 nymphs in ethanol [KLHC].

Algeria, Kabylia, Assif d’Ath Agad (A2), 36°30.26'N, 4°11.93'E, 510 m, 19 March 2017; 10 nymphs in ethanol [KLHC] • same locality, 24 May 2017; 3 nymphs in ethanol [KLHC] • same locality, 30 April 2018; 7 nymphs in ethanol [KLHC].

Algeria, Kabylia, Assif Larbâa (A4), 36°31.07'N, 4°12.07'E, 380 m, 19 March 2017; 4 nymphs in ethanol [KLHC] • same locality, 24 May 2017; 6 nymphs in ethanol [KLHC] • same locality, 30 April 2018; 5 nymphs in ethanol [KLHC].

Algeria, Kabylia, Assif Sahel, Com. de Ath zikki (SA1), 36°32.71'N, 4°29.58'E, 1200 m, 21 April 2017; 32 nymphs in ethanol [KLHC] • same locality, 30 May 2018; 15 nymphs in ethanol [KLHC].

Algeria, Kabylia, Assif Sahel (SA2), 36°32.78'N, 4°29.58'E, 1140 m, 21 April 2017; 11 nymphs in ethanol [KLHC] • same locality, 30 May 2018; 8 nymphs in ethanol [KLHC].

Algeria, Kabylia, Assif Sahel (SA3), 36°35.37'N, 4°27.57'E, 430 m, 21 April 2017; 5 nymphs in ethanol [KLHC] • same locality, 30 March 2018; 3 nymphs in ethanol [KLHC].

Algeria, Kabylia, Assif Chemlili, Com. Boghni (TG2), 36°28.27'N, 3°59.84'E, 1250 m, 25 May 2018; 8 nymphs in ethanol (GBIFCH00835059) ; 2 nymphs on slide, (GBIFCH00673194–GBIFCH00673195) [MZL] • same locality, 1 June 2016; 29 nymphs in ethanol [KLHC] • same locality, 15 March 2017; 10 nymphs in ethanol [KLHC] • same locality, 29 April 2017; 30 nymphs in ethanol [KLHC] • same locality, 2 June 2017; 18 nymphs in ethanol [KLHC] • same locality, 25 May 2018; 38 nymphs in ethanol [KLHC] • same locality, 12 June 2019; 7 nymphs in ethanol [KLHC].

Algeria, Kabylia, Assif Chemlili (TG1), 36°28.32'N, 4°00.16'E, 1450 m, 1 June 2016; 28 nymphs in ethanol [KLHC] • same locality, 15 March 2017; 10 nymphs in ethanol [KLHC]. All L.H. Kechemir coll.

#### Description.

**Male imago.** Size: body length: 6.5 mm; forewing length: 7 mm; cerci and terminal filament length: 8.2 mm.

**Figure 2.**
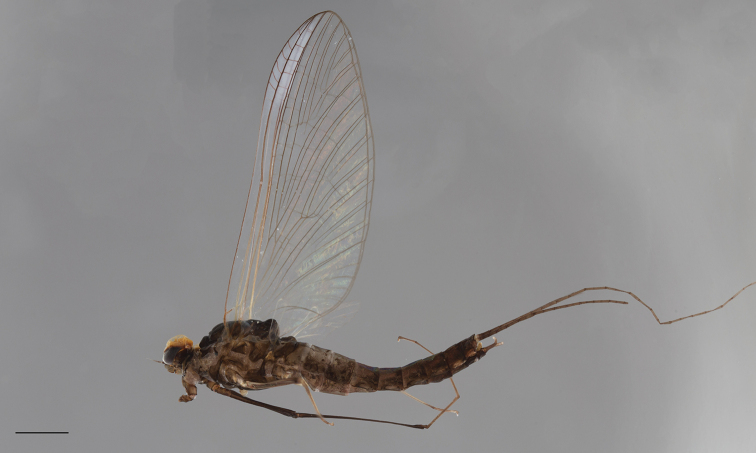
*Habrophlebia djurdjurensis* sp. nov., male imago in lateral view. Scale bar: 1 mm.

***Head*** medium brown, dark brown between ocelli; basal portion of compound eyes greyish, upper portion orange brown (Fig. 2), scape medium brown, pedicel dark brown, flagellum light brown.

***Thorax.*** Pronotum greyish brown, washed with dark brown; meso- and metanotum uniformly dark brown, pleurae, coxae, and trochanters greyish brown, washed with dark brown; fore femora greyish brown, fore tibiae medium brown, tarsi light brown; mid- and hind legs with femora greyish brown on upper surface, tibiae greyish brown in proximal part, light brown in distal part and tarsi medium brown (Fig. 2).

**Figure 3.**
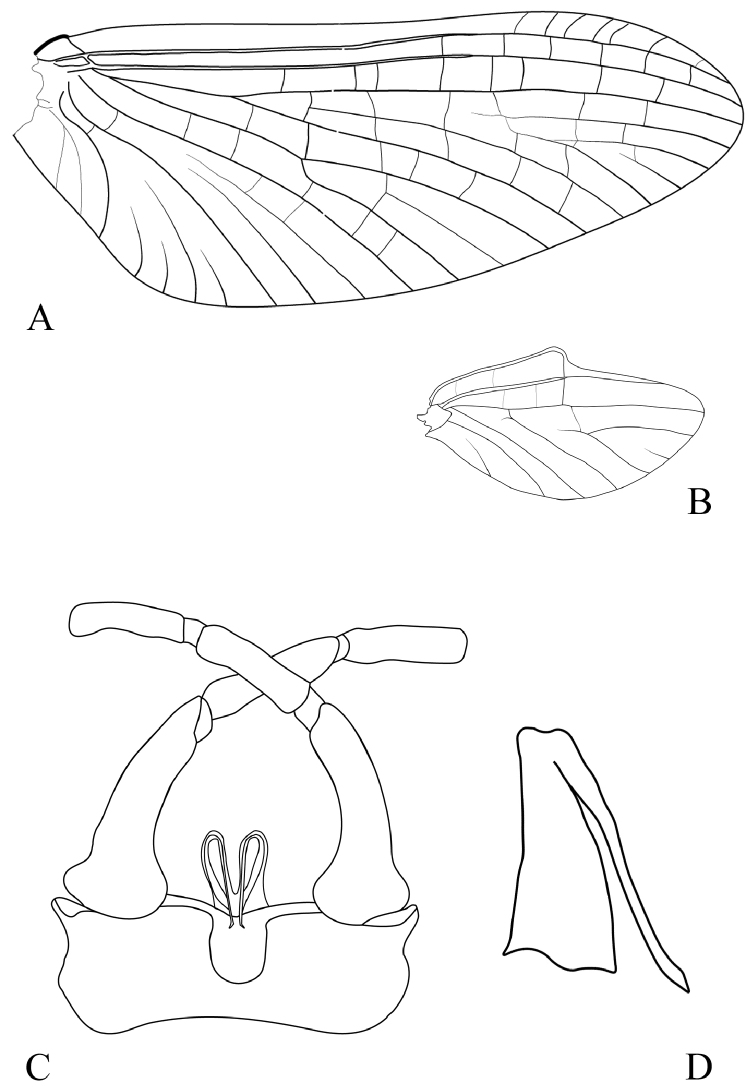
*Habrophlebia djurdjurensis* sp. nov., male imago. **A** Fore wing **B** hind wing **C** genitalia in ventral view **D** penis in lateral view.

Femur/tibia/tarsi ratio in fore leg: 1/0.9/0.2/0.2/0.1/0.1; mid leg: 1/0.8/0.09/0.09/0.05/0.2; hind leg: 1/0.9/0.05/0.04/0.05/0.15. Fore claws similar, paddle-shaped, mid- and hind claws dissimilar, one paddle-like and one hooked. Fore wing (Fig. 3A) transparent, pterostigmatic area milky with ca 7 oblique and simple transversal veins, longitudinal veins light brown, transversal veins whitish. MA and MP forks asymmetrical, cubital field with two short and two long intercalary veins. Hind wing (Fig. 3B) with rounded costal process approximately in the middle of the wing; vein Sc short, not reaching the apex of the wing.

***Abdomen.*** Terga and sterna colorations as in the nymph.

Styliger plate medium brown, first segment of the gonopods greyish brown, segments 2 and 3 yellowish brown. Posterior margin of the styliger plate strongly convex in the middle, median incision regularly rounded, U-shaped (Fig. 3C); segment 1 slightly shorter than segments 2 and 3 combined; ratio length segment 1 vs segment 2: app. 1.5 and segment 1 vs segment 3: app. 1.7; inner margin of segment 1 with a broad base, and a bulge on the outer margin. Penis lobes rounded and well separated from each other, ventral spine long, thin and curved, reaching the base of the styliger plate (Fig. 3D)

Cerci and terminal filament light brown, darker at base.

**Figure 4.**
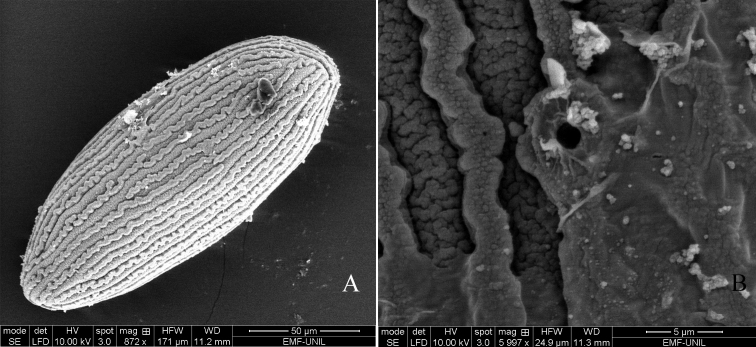
*Habrophlebia djurdjurensis* sp. nov, egg. **A** View of the general shape **B** details of the chorionic ridges and micropyle.

**Eggs** (extracted from mature female nymphs). General shape ovoid, ca 170 μm × 70 μm (Fig. 4A), chorion covered by long longitudinal ribs almost running from one pole to the other, less than 3 μm wide, entire, without punctuation; micropyle in equatorial area (Fig. 4B).

**Figure 5.**
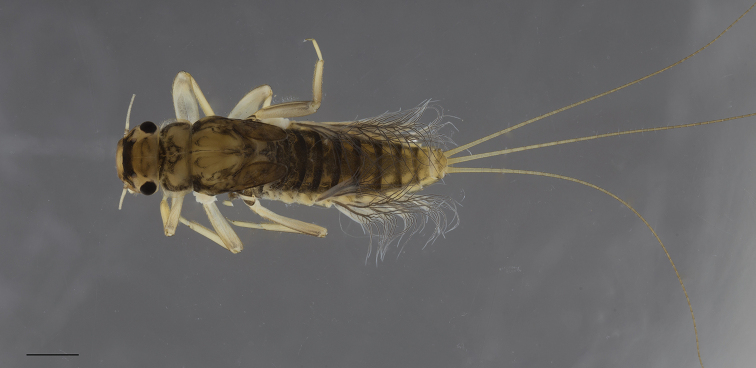
*Habrophlebia djurdjurensis* sp. nov., nymph in dorsal view. Scale bar: 1 mm.

**Nymph.** Body length of final instar, excluding caudal filaments, 5.3–6.3 mm for male and 7.2– 10 mm for female. Cerci longer than body length. General coloration dark brown with light brown markings mainly on abdominal terga (Fig. 5).

**Figure 6.**
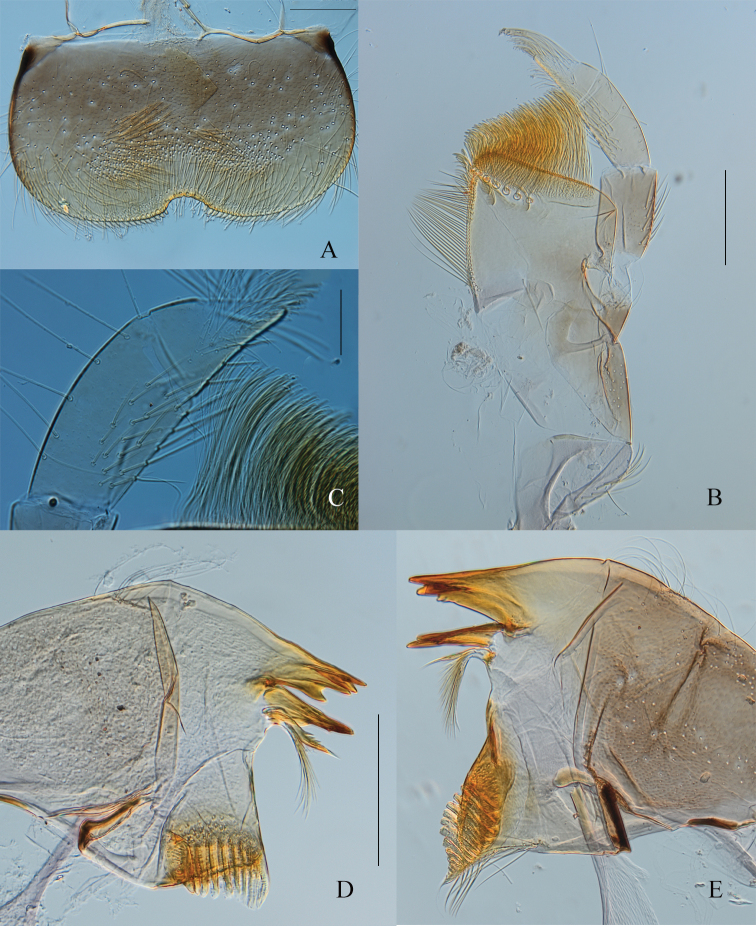
*Habrophlebia djurdjurensis* sp. nov., nymphal mouthparts: **A** labrum in dorsal view **B** maxilla **C** apex of maxillary palp **D** right mandible in dorsal view **E** left mandible in dorsal view. Scale bars: 50 μm (**A**); 200 μm (**B–E**).

**Figure 7.**
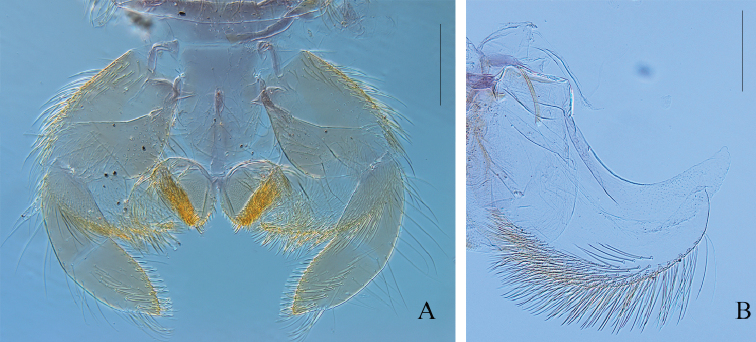
*Habrophlebia djurdjurensis* sp. nov., nymphal mouthparts. **A** Labium in ventral view **B** hypopharynx (left half). Scale bars: 200 μm.

***Head.*** General coloration medium brown; paler area between compound eyes and lateral ocelli; between ocelli, a large dark-brown mark not reaching the clypeus distally, and extending laterally in front of the compound eyes; vertex sutures yellowish, well visible. Upper portion of male eyes orange-brown. Antenna with scape and pedicel greyish brown, filament whitish. Labrum (Fig. 6A) rectangular, wider than long; dorsal surface covered distally with scattered stout setae, proximally with long and thin setae; anterior margin with a row of stout and spatulate setae; anteromedian emargination narrow with four flat/rounded denticles; ventral surface with two bunches of stout setae medially. Maxilla (Fig. 6B) stocky, subapical row of 6 or 7 pectinate setae; maxillary palp 3-segmented, segment 1 as long as segment 2, and longer than segment 3; segment 3 triangular app. 1.7× longer than wide at base; all stout setae on the palp entire, none feathered (Fig. 6C). Mandibles similar to other *Habrophlebia* species (Fig. 6D, E). Hypopharynx with developed superlinguae ending with a small membranous digitation (Fig. 7B). Labium (Fig. 7A) with glossae rhomboid, outer margin and apex covered with stout and short setae; paraglossae enlarged laterally, covered with thin and long setae on dorsal surface; with stout and long setae on outer margin; labial palp 3-segmented; inner margin of segment 1 greatly enlarged towards apex, ca 0.8× longer than maximum width, segments 2 and 3 subequal in length, ca 0.7× length of segment 1; segment 3 ca 1.2× longer than wide at base and slightly triangular.

**Figure 8.**
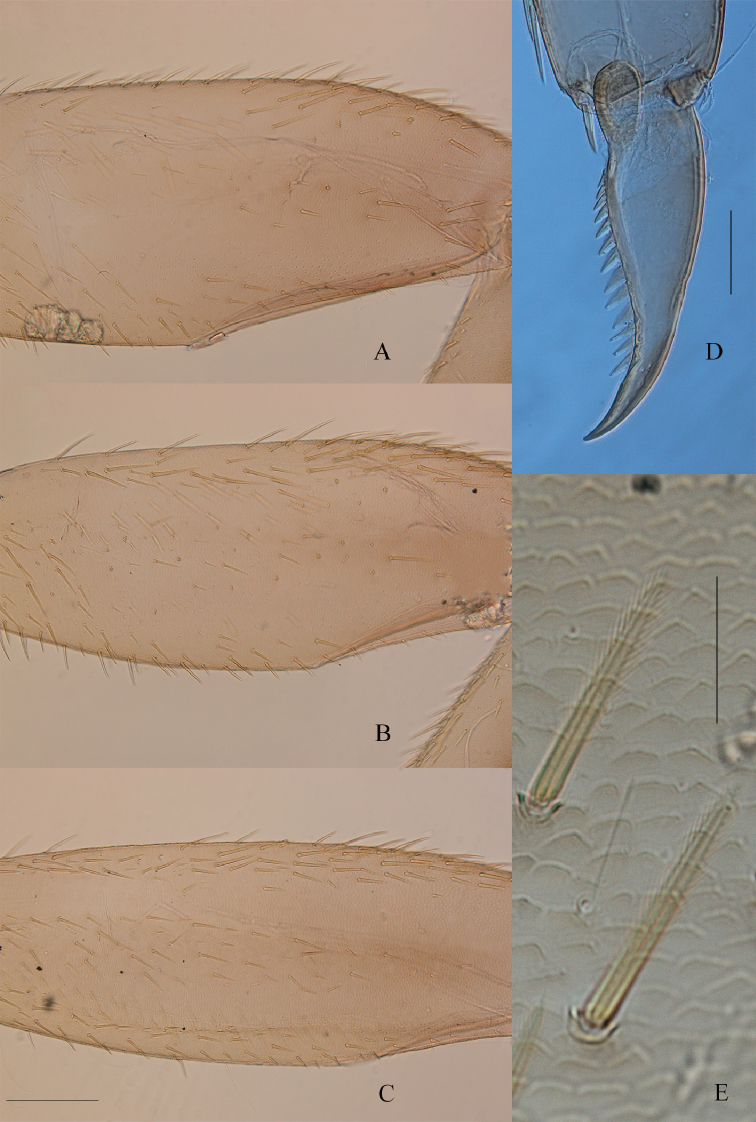
*Habrophlebia djurdjurensis* sp. nov., nymphal legs. **A** Fore femur **B** middle femur **C** hind femur **D** tarsal claw **E** feathered setae on the dorsal face of hind femur. Scale bars: 200 μm (**A–C**); 50 μm (**D**); 30 μm (**E**).

***Thorax.*** Pro- and mesonotum greyish brown, with black maculae, especially on lateral margins (Fig. 5). Legs yellowish brown to medium brown; dorsal surface of femora almost entirely washed with greyish brown macula; tarsi and tibiae generally lighter, except sometimes in mature nymphs. Anterolateral angles of each hemi-pronotum with a bunch of long setae, anterior margin with a single row of long setae not reaching the median suture. Fore femora (Fig. 8A) elongated, ca 2.3 longer than wide, upper surface covered with long, entire, and pointed setae; fore tibiae subequal in length to femora, outer margin with thin and long setae, inner margin with several rows of long stout and pointed setae not feathered; tarsi 0.8× length of tibiae, outer margin with long and thin setae, inner margin with two rows of long, stout, pointed setae. Middle legs (Fig. 8B) similar to fore legs, dorsal surface of femora with more numerous and slightly longer stout and pointed setae; tibiae 0.8× length of femora and tarsi 0.5× length of tibiae. Hind femora (Fig. 8C) 3× longer than wide, dorsal surface covered with stout, long, pointed and feathered setae (only visible at high magnification: 400× and more; Fig. 8F); ventral surface with few feathered setae; hind tibiae as long as hind femora, outer margin with scattered stout, pointed setae; inner margin with several rows of stout, pointed, entire setae; tarsi 0.4× length of tibiae, outer margin with long and thin setae, inner margin with two rows of long, stout, pointed setae. Claws (Fig. 8D) of all legs slightly hooked, with 15–18 long, thin, pointed denticles including three larger towards the middle of the claws.

**Figure 9.**
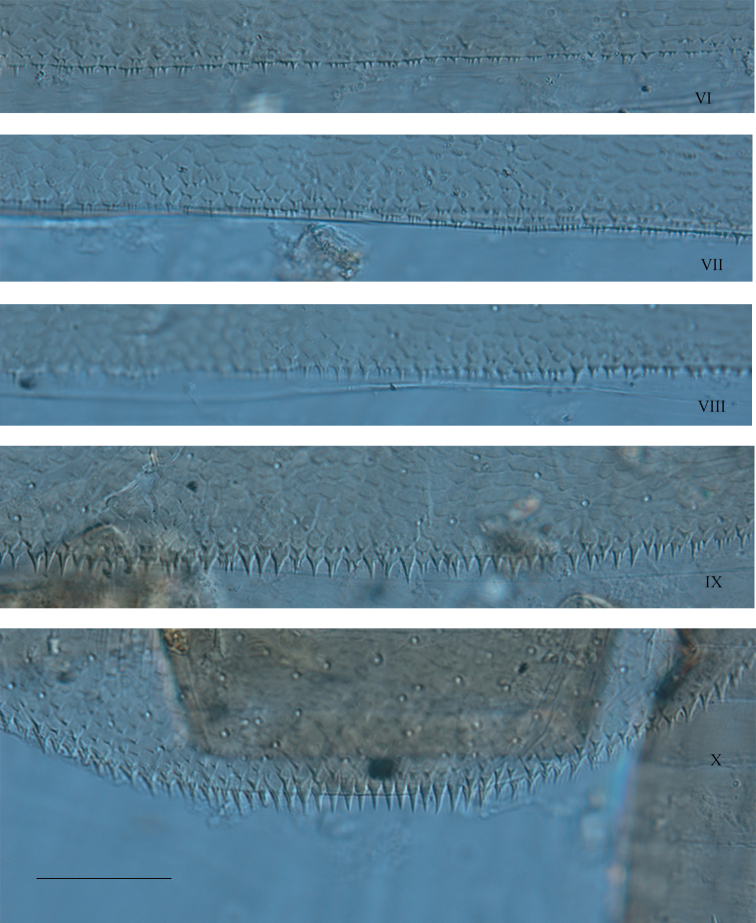
*Habrophlebia djurdjurensis* sp. nov., posterior margin of abdominal terga VI–X. Scale bar: 50 μm.

**Figure 10.**
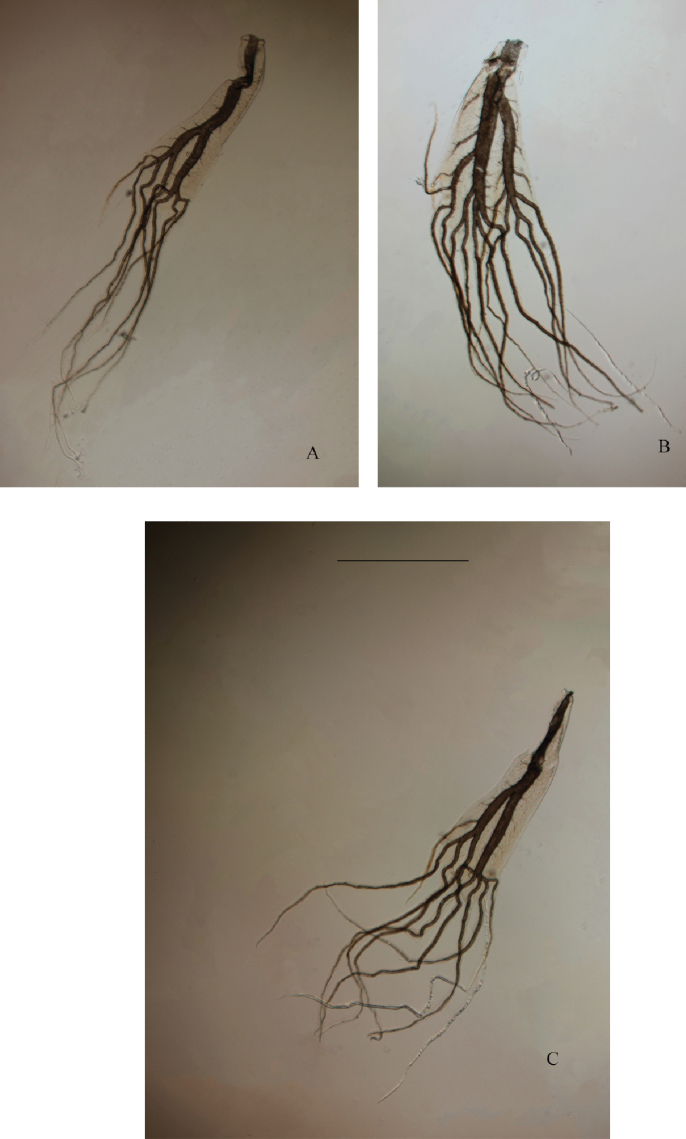
*Habrophlebia djurdjurensis* sp. nov., nymphal gills. **A** Gill I **B** gill IV **C** gill VII. Scale bar: 0.5 mm.

***Abdomen.*** Terga greyish brown to dark brown with characteristic light markings (Fig. 5). Terga I–II dark brown, terga II–IX with two sublateral elongated dark brown maculae, joining on the posterior margin, leaving two lateral light areas inconspicuous on terga III–IV, more pronounced on terga V–IX; terga X light brown. Sternum I entirely greyish brown; sterna II–IX greyish brown with lateral and anterior bands light brown; nervous ganglia greyish brown. Posterolateral expansions only on segments VIII and IX. Ornamentation of the terga (Fig. 9): posterior margin of tergum X with well developed, narrow, pointed spines, ca 3× longer than wide; tergum IX with narrower and shorter spines, bordered with submarginal microdenticles; tergum VIII with minute spines, a little bit longer than the submarginal microdenticles; terga V–VII with tiny spines; terga I–IV with barely visible spines at high magnification (400×). Gills present on segments I–VII; all gills long and large; first gill (Fig. 10A) with ventral lamella bearing 3 or 4 filaments, upper lamella with 4 or 5 filaments, gills II–VI (Fig. 10B) with 4–7 and 8–11 filaments on the ventral and dorsal lamella respectively, gill VII (Fig. 10C) with about 6 filaments on ventral lamella and ca 9 filaments on upper lamella.

Cerci and terminal filament yellowish brown, medium brown in mature nymphs.

#### Etymology.

The species is named after the Kabylian Massif of Djurdjura.

#### Affinities.

The male imago of *Habrophlebia djurdjurensis* sp. nov. can be easily separated from that of *H. antoninoi* by the shape of the hind wing, from *H. hassainae* by the length of vein Sc on the hind wing, from *H. antoninoi*, *H. consiglioi*, *H. eldae*, *H. fusca*, *H. lauta*, and *H. vaillantorum* by the shape of the notch of the stilyger which is more or less narrowed, from *H. consiglioi* by the shape of the bulge on the outer margin of the first segment of the gonopods, from *H. consiglioi* and *H. antoninoi* by the shape of the penial spine which is not stout, and from *H. vaillantorum*, *H. fusca*, and *H. lauta* by penis lobes in ventral view which are narrow and hold tight against each other with penial spine longer and curved at the apex. The new species is more similar to *H. lauta*, with whom it shares the shape of penial spine which is long, thin, and more pointed, and related with *H. hassainae*, with whom it shares the styliger shape but differs by the length of the penial spine much shorter.

The nymph of *H. djurdjurensis* differs from those of *H. consiglioi*, *H. eldae*, *H. fusca*, and *H. vaillantorum* by the pattern of coloration on the abdomen, from *H eldae*, *H. fusca*, and *H. lauta* by the shape of the superlingua of the hypopharynx, and from all other species by the shape of the spines on the posterior margin of the terga. Pronotum ornamentation is similar to *H. hassainae*, somewhat intermediate between *H. consiglioi* and *H. eldae* (see Belfiore and Gaino 1985: figs 5, 6). The ventral surface of hind femora bears very few setae compared to *H. hassainae*. The nymph is most similar to that of *H. vaillantorum*, from which it can be separated by the abdomen color, the ornamentation of terga (especially tergum IX), the number and the length of filaments on gills which are longer in *H. djurdjurensis* compared to *H. vaillantorum*.

Among all Palaearctic species of *Habrophlebia*, *H. djurdjurensis* is the second species after *H. hassainae* with the greatest number of denticles on claws (15–18 denticles vs 11–16 in others and 18–22 in *H. hassainae*). It possesses gills with the greatest number of filaments on each lamella still after *H. hassainae* (4–7 in ventral and 8–11 in dorsal, vs 1–6 and 3–9 in ventral and dorsal lamella, respectively in all other species, and 5–8 and 9–12 in ventral and dorsal lamella, respectively, in *H. hassainae*).

The eggs of *H. djurdjurensis* have a length/width ratio of ca 2.4, intermediate between those of *H. lauta* (2.7), *H. hassainae* (2.1), and *H. vaillantorum* (1.7). These eggs are also relatively smaller than the others; compared to *H. hassainae*, the longitudinal ribs are longer reaching almost from one pole to the other, whereas in the latter, two to three ribs are necessary to reach both poles.

#### Distribution and ecology.

The genus *Habrophlebia* currently encompasses eight species in the Western Palearctic region. Two species, *H. lauta* McLachlan, 1884 and *H. fusca* (Curtis, 1834), are widely distributed in Europe. *Habrophlebia eldae* Jacob & Sartori, 1984 was considered a Mediterranean element, which recently expanded its geographic range to central Europe, probably due to global climate change (Wagner et al. 2007). *Habrophlebia consiglioi* was described from the island of Sardinia and later reported from Tunisia (Zrelli et al. 2011). The others have a much restricted distribution: *H. antoninoi* is only known from two locality near Andújar in southern Spain (Alba-Tercedor 2000), *H. vaillantorum* is probably endemic to the Moroccan High Atlas (Thomas et al. 1999), and *H. hassainae* has not been reported yet outside of the Tafna watershed (western Algeria). The presence of *H. djurdjurensis* for now seems to be limited to the Kabylian massif of Djurdjura, and the species may be a microendemic to this region of Algeria. Unpublished data from other parts of Algeria suggest this species is not found in those areas. *Habrophlebia djurdjurensis* has a rithrophilic tendency and is widely distributed in the streams on the north slopes of the Djurdjura. It colonizes varied biotopes, from spring streams to foothill rivers. It was collected in 19 stations between 300 and 1450 m above sea level (Fig. .1). The habitats are characterized by a mixed substrate of pebbles, gravel, sand, and silts covered in some places by debris, moderate currents, and water temperatures not exceeding 20 °C (Table 1). This species appears unable to endure warmer waters at low altitudes and quickly disappears from stations below 380 m.

### 

**Table 1.** Geographical and physical data of sampling sites of Great Kabylia basin Algeria (sampling sites with *H. djurdjurensis*).

Sites	Latitude N/ Longitude E	Altitude (m)	Orientation	Distance from the source (km)	Width of riverbed (m)	Dominant Substrate	Turbidity	Maximum depth (cm)	Riparian vegetation
SA1	36°32.712'N, 004°29.575'E	1200	S-N	1.5	1.5	Sh, G	C	20	Ps
SA2	36°32.782'N, 004°29.557'E	1140	S-N	2	1.5	Sh, G,Vd	C	30	Ps
SA3	36°35.368'N, 004°27.572'E	430	S-N	6	2	Sh, G, S	T	30	Ps
TR1	36°29.4305'N, 004°21.693'E	1200	S-N	0.5	1.3	Sh, G, S	C	50	Ps
TR2	36°29.430'N, 004°21.534'E	1150	S-N	1.5	0.7	Sh, G, Si	C	20	Ps, Th
AA	36°29.710'N, 004°22.382'E	1080	S-N	0.5	1	Sh, G, S	C	20	Ps, Th
AI	36°30.412'N, 004°24.075'E	1010	S-N	1	1	Sh, G,	C	20	Ps, Th
D1	36°30.381'N, 004°19.937'E	900	S-N	0.5	1	Sh, G, S, Si, Vd	C	20	Ps
A1	36°29.683'N, 004°11.136'E	600	S-N	0.5	0.8	Sh, G,Vd	C	30	Ps
A2	36°30.260'N, 004°11.930'E	510	S-N	1	0.5	Sh, G,S,Vd	C	10	Ps
A4	36°31.066'N, 004°12.073'E	380	S-N	4.5	3	Sh, G, S	T	50	Hb, Th
O1	36°29.976'N, 004°03.931'E	800	S-N	2.5	2	Sh, G	C	30	Ps
O3	36°29.279'N, 004°07.362'E	1040	S-N	0.8	0.5	Sh, G	C	20	Ps, Th
O4	36°29.482'N, 004°07.489'E	950	S-N	1.5	1.8	Sh, G	C	30	Ps
O5	36°30.723'N, 004°06.666'E	500	S-N	13	3	Sh, S, Vd	C	30	Ps
O6	36°31.877'N, 004°06.848'E	290	S-N	20	3.5	Sh, G, S, Si	T	40	Ps, Th
TG1	36°28.320'N, 004°00.160'E	1450	S-N	1	0.7	Sh, G,Vd	C	10	Hb, Th
TG2	36°28.267'N, 003°59.84'E	1250	S-N	0.7	1	Sh, G,Vd	C	10	Ps
TG3	36°28.049'N, 003°58.308'E	900	S-N	0.5	0.8	Sh, G,Vd	C	15	Ps

Sh= shingle, G = gravels, S = sands, Si = silts, Vd= Vegetal debris, C = clear, T = turbid, Ps = pluristratified, Hb = herbaceous, Th= thorny.

## Supplementary Material

XML Treatment for Habrophlebia djurdjurensis
